# Knowledge and attitude towards sickle cell anemia among care givers of paediatric sickle cell patients at a tertiary hospital in Eastern Uganda: a cross sectional study

**DOI:** 10.1186/s13104-023-06633-3

**Published:** 2023-11-27

**Authors:** Christine H Namugerwa, Yahaya Gavamukulya, Banson John Barugahare

**Affiliations:** 1 Department of Nursing, Faculty of Health Sciences, Busitema University, P.O. Box 1460, Mbale, Uganda; 2 Department of Biochemistry and Molecular Biology, Faculty of Health Sciences, Busitema University, P.O. Box 1460, Mbale, Uganda; 3 Department of Microbiology and Immunology, Faculty of Health Sciences, Busitema University, P.O. Box 1460, Mbale, Uganda

**Keywords:** Sickle cell anemia, Sickle cell Disease, Knowledge, Attitude, Eastern Uganda

## Abstract

**Objective:**

To explore the knowledge and attitude towards sickle cell disease (SCD) among care givers of paediatric sickle cell patients at Mbale regional referral hospital in Eastern Uganda.

**Methods:**

A cross sectional study was conducted at Mbale regional referral hospital. We used simple random sampling technique to recruit participants from among the care givers of pediatric sickle cell patients admitted at the hospital, administered questionnaires and conducted multivariable logistic regression to establish the association between the different factors.

**Results:**

372 respondents participated in the study, 82.26% of which were female. 57.80% of the respondents had ever heard of SCD/SCA. 36.02% were willing to stay in a relationship with their partner despite the risk of having a child with SCD/SCA. A multivariate analysis revealed that variables; “number of children”, “children with sickle cell can cope with life” and “willing to stay in a relationship despite the risk of a having a child with sickle cell” were statistically significant.

**Conclusion:**

There was a high level of general awareness about SCD/SCA but comprehensive knowledge about its cause and prevention was low and the majority did not find a reason as to why it should influence their marital decisions. Inclusion of SCD/SCA in existing health education programs is highly recommended.

**Supplementary Information:**

The online version contains supplementary material available at 10.1186/s13104-023-06633-3.

## Introduction

Sickle cell disease is a group of genetic disorders characterized by the inheritance of sickle hemoglobin (Hb S) from both parents or Hb S from one parent and a gene for an abnormal hemoglobin or β-thalassemia from the other parent [1]. The presence of Hb S can cause red blood cells to change from their usual biconcave disc shape to a crescent or sickle shape during de-oxygenation. Upon re-oxygenation, the red blood cell regains a normal configuration, but after repeated cycles of “sickling and un-sickling,” the erythrocyte becomes damaged permanently and may remain sickled or may hemolyze. This hemolysis is responsible for the anemia that is the hallmark of sickle cell disease [2].

Sickle-cell anemia is particularly common among people whose ancestors come from sub-Saharan Africa, India, Saudi Arabia and Mediterranean countries. In some areas of sub-Saharan Africa, up to 2% of all children are born with the condition. In broad terms, the prevalence of the sickle-cell trait (healthy carriers who have inherited the mutant gene from only one parent) ranges between 10% and 40% across equatorial Africa and decreases to between 1% and 2% in the north African coast and < 1% in South Africa. In West African countries such as Ghana and Nigeria, the frequency of the trait is 15–30% whereas in East African countries, Uganda has the highest rate of SCD about 45% [3]. In Uganda it shows marked tribal variations, Northern Uganda has the highest prevalence of Sickle cell trait in the country standing at 18.6%, East-Central regions come second with a sickle cell prevalence of 16.7%, the Mid-Eastern and South-Western regions have sickle cell trait prevalence of 16.5% and of 4.1% respectively [4]. Children with sickle cell disease may display physical manifestations of their illness. As a result of short stature, low muscle mass or jaundiced eyes and nail beds, ridicule by peers and others is possible. This is particularly common in children below 14 years [5]. This calls for a proper education and sensitization of parents, peers and communities about their condition and status.

There is still a gap in the knowledge about SCA/ SCD among very many people. A study reported that uptake of sickle cell trait screening services was low among university students and therefore the need for the service to be encouraged among students at universities [6]. Similarly, another study reported that parents do not know much about the disease and how best to look after their children with SCA [7]. Children and their parents should be prepared to use coping strategies to help them in these situations. This study therefore sought to explore the knowledge and attitude towards sickle cell disease /anemia among caregivers of paediatric sickle cell patients at Mbale regional referral hospital in Eastern Uganda.

## Materials and methods

### Study design and area

A cross sectional study design was used among caregivers of pediatric sickle cell patients at Mbale regional referral hospital. Mbale regional referral hospital is located in the Eastern Uganda in Mbale district. It is a referral hospital for districts of Busia, Budaka, Butebo, Kibuku, Kapchorwa, Bukwo, Butaleja, Manafwa, Mbale and Pallisa.

### Study population and sampling technique

The target populations were the caregivers of pediatric sickle cell patients at MRRH. All caregivers that attended to pediatric sickle cell patients at MRRH, but a minimum sample size was calculated using Leslie Kish formula [8]. Prevalence estimates of sample size was calculated based on findings from a similar study which showed a prevalence of 73% [9]. Using 95% confidence level and 5% margin error, a required sample size of 363 respondents was obtained. Participants were selected using random sampling technique.

### Inclusion and exclusion criteria

Inclusion: all caregivers above 18 years of age and had consented to participate in the study.

Exclusion: all caregivers who did not consent to participate in the study, were absent, deaf or blind.

### Data collection method

An English interviewer online administered questionnaire (Supplementary file 1), accessible at, https://ee.kobotoolbox.org/x/6G5hPgud, consisting of 25 questions was used as the major tool for data collection. The replies of the respondents were directly filled into the online questionnaire by the researcher. A language best understood by the respondent was used and effective communication was maintained.

### Operational definitions

A caregiver by definition as per this study is a person who tends to the needs or concerns of a child with sickle cell disease.

Knowledge of sickle cell anemia was determined by 10 questions from the questionnaire that focused on determining the knowledge that the caregivers had about sickle cell disease/ sickle cell anemia. Having good knowledge was considered as answering more than 5 questions correctly and having poor knowledge was considered answering less than 5 questions correctly.

Attitude of care givers towards sickle cell anemia was assessed using 6 questions from the questionnaire; and a care giver was considered to have good attitude when 5 of the 6 questions were answered correctly and considered to have bad attitude when 1 or none of the 6 questions were answered correctly.

### Data quality control

The questionnaire was pretested at the pediatric unit and sickle cell clinic of MRRH randomly before use ascertaining its reliability and validity.

### Data analysis

The responses to different questions were tabulated according to the numbers of caregivers who choose the different options provided for open ended questions and those who gave correct and wrong options to the closed ended questions. The data was then analyzed using STATA and Microsoft spread sheet and presented using pie charts and bar graphs.

### Study limitation

The findings may not be generalized to the whole community at large as it only involved caregivers who came to the health facility to care for patients. Only caregivers with sickle cell patients were considered respondents hence views about knowledge and attitude of other people were not recorded or considered in the study.

## Results

### Socio-demographic characteristics of respondents

A total of 372 respondents participated in the study of which 306 (82.26%) were females and 17.74% males. The major age was that of 31 years to 40 years with 164(44.09%) and more than half of the respondents, 200(53.76%), were of primary education level. The highest number of respondents, 262(70.43%), had a total number 5 children or less, with more than half of the respondents, 263(70.70%), having at least a child with sickle cell disease as shown in Table 1.

**Table 1:** Socio-demographic characteristics of respondents

Variable	Frequency(N = 372)	Percentage (%)
**Gender**		
Female	306	82.26
Male	66	17.74
**Age**		
18 to 30	112	30.11
31 to 40	164	44.09
41 to 50	84	22.58
> 50	12	3.23
**Religion**		
SDA	13	3.49
Born-again	48	12.90
Catholic	103	27.69
Moslem	85	22.85
Protestant	121	32.53
Others	2	0.54
**Education background**		
Primary	200	53.76
Secondary	111	29.84
tertiary	26	6.99
uneducated	35	9.41
**Occupation**		
Employed	118	31.72
unemployed	254	68.28
**Marital status**		
Married	314	84.41
Not married	58	15.59
**Number of children**		
<=5	262	70.43
> 5	110	29.57
**Number of children with SCD/SCA**		
1	263	70.70
> 1	109	29.30

### Knowledge on sickle cell anemia/ sickle cell disease

More than half of the respondents (57.80%) had ever heard about sickle cell and 292 (78.49%) respondents did not know how a person could acquire it. Of the 80 (21.51%) respondents that knew how a person could get SCD, 55 (68.75%) respondents knew it is got from parents. 350 (94.09%) of the respondents knew about sickle crisis of which the commonest symptoms were pain (74.19%), jaundice (53.49%), and swelling of hands and feet (59.95%). More than half of the respondents (69.89%) agreed that child had to take medication and be monitored daily so as to prevent complications as shown in Table 2.

**Table 2:** Knowledge on SCA/SCD

Variable	Frequency (N = 372)	Percentage (%)
**Heard about SCA/SCD before**		
No	157	42.20
Yes	215	57.80
**Know how a person can get SCD**		
No	292	78.49
Yes	80	21.51
**Sickle cell transmission. (N = 80)**		
Parents	55	68.75
i don’t know	6	7.50
Others	19	23.75
**Others, specify; n = 19**		
Cursed	7	36.84
Witchcraft	9	47.37
Mother	3	15.79
**Have any other person in your family with sickle cell**		
Yes	163	43.82
No	75	20.16
i don’t know	134	36.02
**If yes, specify who**		
Maternal	65	39.88
Paternal	98	60.12
**Know sickle cell crisis**		
No	22	5.91
Yes	350	94.09
**Signs and symptoms of sickle cell (N = 372)**		
Pain	276	74.19
Fever	136	36.56
Fatigue	80	21.52
Jaundice	199	53.49
Headache	98	26.34
Swelling of hands and feet	223	59.95
Others	29	7.80
**To prevent complications child must take medicine and be monitored daily**		
Agree	260	69.89
Disagree	53	14.25
i don’t know	59	15.86
**Tests for sickle cell**		
blood test	335	90.68
don’t know	36	9.68
urine test	12	3.23

### Level of knowledge about SCD/ SCA among participants

**Fig. 1:**
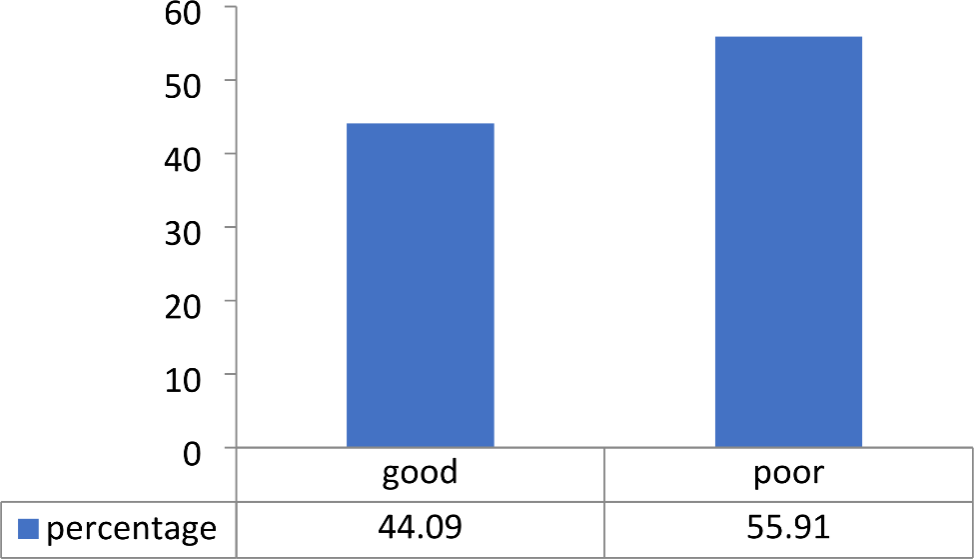
Level of knowledge about SCD/SCA among participants

The study revealed that 55.91% of the participants had poor knowledge about sickle cell disease, whereas 44.09% had good knowledge about the disease as shown in Fig. 1.

### Respondents’ attitude towards SCA/SCD

Majority of the respondents 193 (51.88%) did not know if sickle cell can be cured an unsurprisingly, 134(36.02%) respondents were willing to stay in a relationship despite the risk of having a child with SCD/SCA. While 305 (81.99%) respondents were sad about having a child with sickle cell, the majority of the respondents (90.59%) were not aware of the existence of social networks for children with sickle cell as shown in Table 3.

**Table 3:** Respondents’ attitude towards SCA/SCD

Variable	Frequency (N = 372)	Percentage (%)	
**Sickle cell can be cured**			
Agree	86	23.12	
disagree	93	25	
I don’t know	193	51.88	
**If agree, why? (N = 86)**			
God	40	46.51	
medicine	4	4.65	
People	36	41.86	
Seen	6	6.98	
**Children with sickle cell can cope with life**			
Agree	179	48.12	
disagree	101	27.15	
I don’t know	92	24.73	
**Willing to stay in a relationship with your partner despite the risk of having a child with sickle cell**			
Yes	134	36.02	
No	109	29.30	
Maybe	129	34.68	
**How you feel about having a child with sickle cell**			
Sad	305	81.99	
Tiresome	44	11.83	
Cursed	16	4.30	
Strong	3	0.81	
don’t know	4	1.08	
**Aware that there social networks for children with sickle cell**			
No, I don’t know of any	337	90.59	
Yes I know, and I am in contact with one	4	1.08	
Yes I know, and am not in contact with any	30	8.06	
Others	1	0.27	

### Attitude of caregivers towards sickle cell disease

**Fig. 2:**
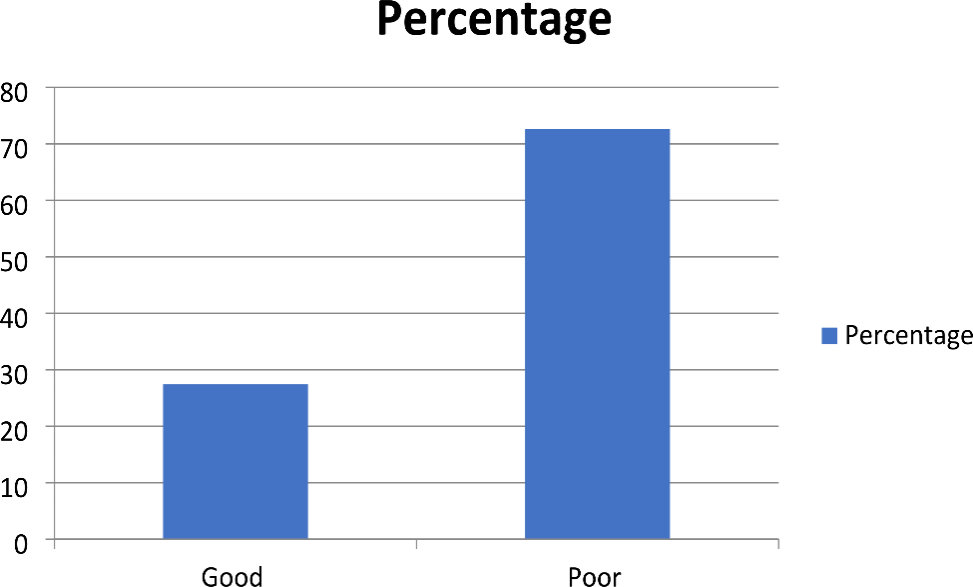
Attitude of caregivers towards sickle cell anemia

As shown in Fig. 2, majority of participants (72.58%) had a poor attitude towards sickle cell disease, whereas 27.42% had good attitude towards sickle cell interventions and health seeking behavior.

### Association between socio demographic factors and knowledge

The analysis showed that the socio demographic factors significantly associated with knowledge were variables; number of children and number of children with SCA/SCD having a p-value < 0.05, as shown in Table 4.

**Table 4:** The association between socio demographic variables and knowledge among caregivers of pediatric sickle cell patients in Mbale Regional Referral Hospital

Knowledge, n (%)				
Variable	Good	Poor	Total	P value
**Gender**				
Female	132 80.49	174 83.65	306 82.26	0.427
Male	32 19.51	34 16.35	66 17.74	
**Age**				
18 to 30	45 27.44	67 32.21	112 30.11	0.142
31 to 40	67 40.85	97 46.63	164 44.09	
41 to 50	45 27.44	39 18.75	84 22.58	
Above 50	7 4.27	5 2.40	12 3.23	
**Religion**				
Protestant	56 34.15	65 31.25	121 32.53	0.676
Catholic	41 25.00	62 29.81	103 27.69	
Moslem	38 23.17	47 22.60	85 22.85	
Born again	22 13.41	26 12.50	48 12.90	
SDA	7 4.27	6 2.88	13 3.49	
Others	0 0.00	2 0.96	2 0.54	
**Education background**				
Primary	79 48.17	121 58.17	200 53.76	0.256
Secondary	57 34.76	54 25.96	111 29.84	
Tertiary	7 4.27	6 2.88	13 3.49	
Uneducated	14 8.54	21 10.10	35 9.41	
University	7 4.27	6 2.88	13 3.49	
**Occupation**				
Unemployed	106 64.63	148 71.15	254 68.28	0.354
Self employed	36 21.95	40 19.23	76 20.43	
Formal employed	22 13.41	20 9.62	42 11.29	
**Marital status**				
Married	139 84.76	174 83.65	313 84.14	0.722
Single	13 7.93	21 10.10	34 9.14	
Divorced	10 6.10	9 4.33	19 5.11	
Others	2 1.22	4 1.92	6 1.61	
**Number of children**				
< 5	102 37.80	160 23.08	262 70.43	**0.002**
> 5	62 37.80	48 23.08	110 29.57	
**Number of children with sickle cell**				
1	106 64.63	157 75.48	263 70.70	**0.041**
> 1	58 35.37	50 24.04	108 29.03	

*Note:* The bold fonts were to highlight the key categories of fields shared in the table. It also indicates some values which are of significant interest like where the p value is less than 0.05

### Association between attitude towards sickle cell and knowledge

Factors related to attitude that significantly associated with knowledge towards SCD/SCA were variables; sickle cell can cure, children with sickle cell can cope with life, willing to stay in a relationship despite risk of having a child with sickle cell, aware about social networks for sickle cell having a P value < 0.05 as shown in Table 5.

**Table 5:** The association between attitude towards SCA and knowledge among caregivers of pediatric sickle cell patients at Mbale Regional Referral Hospital

Knowledge, n (%)				
Variable	Good	Poor	Total	P value
**Sickle cell can cure**				
Agree	34 20.73	41 19.71	75 20.16	**0.000**
Disagree	63 38.41	41 19.71	104 27.96	
Don’t know	67 40.85	126 60.58	193 51.88	
**Children with sickle cell can cope with life**				
Agree	93 56.71	86 41.35	179 48.12	**0.000**
Disagree	47 28.66	54 25.96	101 27.15	
Don’t know	24 14.63	68 32.69	92 24.73	
**Willing to stay in a relationship despite the risk of having a child with sickle cell**				
Yes	54 32.93	80 38.46	134 36.02	**0.023**
No	60 36.59	49	109 29.30	
Maybe	50 30.49	79 37.98	129 34.68	
**Aware about social networks for children with sickle cell**				
No, I don’t know of any	140 85.37	197 94.71	337 90.59	**0.016**
Yes, I know and am not in contact with one	21 12.80	9 4.33	30 8.06	
Yes, I know and in contact with one	21.22	0.962	4 1.08	

*Note:* The bold fonts were to highlight the key categories of fields shared in the table. It also indicates some values which are of significant interest like where the p value is less than 0.05

### Crude odds ratios

The multivariate logistic regression revealed that variables; number of children, children with sickle cell, sickle cell can be cured, children with sickle cell can cope with life and willing to stay in a relationship despite having a child with sickle cell were statistically significant at a p-value < 0.05 at 95%CI. (Table 6)

**Table 6:** Factors associated with knowledge towards sickle cell among caregivers of pediatric sickle cell patients at Mbale Regional Referral Hospital

Variables	COR	P-value	95% CI
**Number of children**			
< 5	0.1	**0.002**	0.3 to 0.8
> 5	*1*	-	-
**Number of children with sickle cell**			
1	1.7	**0.019**	1.1 to 2.7
> 1	*1*	-	-
**Sickle cell can be cured**			
Agree	*1*	-	-
Disagree	0.5	**0.044**	0.3 to 1.0
**Children with sickle cell can cope with life**			
Agree	0.3	**0.000**	0.2 to 0.6
Disagree	0.4	**0.004**	0.2 to 0.7
Don’t know	*1*	-	-
**Willing to stay in a relation despite the risk of having a child with sickle cell**			
Yes	1.8	**0.022**	1.0 to 3.0
No	*1*	-	-
Maybe	1.9	**0.012**	1.2 to 3.2
**Aware of organizations that support children with sickle cell**			
No, I don’t know of any	1.4	0.734	0.2 to 10.1
Yes, I know but am not in contact with any	0.4	0.431	0.1 to 3.5
Yes, I know and am in contact of one	-	-	-
Others	*1*	-	-

*Note:* The bold fonts were to highlight the key categories of fields shared in the table. It also indicates some values which are of significant interest like where the p value is less than 0.05

### Adjusted odds ratios (AOR)

As shown in Table 7, the multivariate logistic regression of AOR revealed that variables; number of children, children with sickle cell can cope with life and willing to stay in a relationship despite the risk of a having a child with sickle cell were statistically significant at a p-value < 0.05 at a 95%CI.

**Table 7:** AOR of factors associated with knowledge towards sickle cell among caregivers of pediatric sickle cell patients at Mbale Regional Referral Hospital

Variables	AOR	P > z	95% CI
Number of children	0.1	**0.012**	0.3 to 0.9
Children with SCD/SCA	0.8	0.247	0.5 to 1.2
Sickle cell can be cured	1.2	0.204	0.9 to 1.6
Children with sickle cell can cope with life	1.6	**0.001**	1.2 to 2.1
Willingness to stay in a relationship despite the risk of having a child with sickle cell	1.1	0.690	0.8 to 1.4
Aware of social networks for children with sickle cell	0.7	**0.037**	0.5 to 1.0

*Note:* The bold fonts were to highlight the key categories of fields shared in the table. It also indicates some values which are of significant interest like where the p value is less than 0.05

## Discussion

A lower proportion of the males (66 respondents, 17.74%) participated in the study as compared to females. This may be due to the fact that most men were away for work or other businesses. The study results are similar to earlier studies, which highlighted that more women utilize medical services, which include the sickle cell clinic at the health facility as compared to men [10]. This may continue to underscore community campaigns since men may be the decision makers in most Ugandan homes.

More than half of the participants were of a primary education background, which may influence their knowledge about sickle cell. Majority of the respondents were as well unemployed which explains why many of them were having a hard time to cope and raise children with sickle cell since there a lot of necessities that come with the role; medication, transport to hospital among others. These findings are in line with earlier studies that revealed that relatives or caregivers with higher educational and occupational attainments (compared with those with lower attainments) experienced significantly lesser financial burden, disruption of family routines, and psychological distress [11]. This provides a better explanation as to why many of the respondents reported feelings of sadness (81.99%) while others said they find it tiresome (11.83%) taking care of a sickle cell child, some called it a curse (4.30%), some said they are strong (0.81%) and the remaining respondents did not know how they actually felt (1.08%).

Majority of the participants had less than 5 children (70.43%) in total and with at least one child suffering from sickle cell. If both parents have sickle cell trait, there is a one in four (25%) chance that any given child could be born with sickle cell anaemia. This study therefore shows that there is a 1.2% chance of a caregiver with less than 5 children to have knowledge about sickle cell (p-value = 0.012 which is significant).

Most of the participants (57.80%) had heard of SCA/SCD, which implies that they knew of its existence. However, majority did not know how a person can get sickle cell while the remaining few who claimed to know; 55 respondents believed it can be got from parents, 7 believed it can be got through curses, 9 said witchcraft, 3 said it can be got from the mother only while the remaining 6 said that they actually didn’t know. These results show a variation in respondent’s beliefs and a bit of misconception because while some of the respondents believed that SCD was natural/genetic, a few believed it was “curse from God” and witch craft [12].

Majority of the respondents knew about the signs and symptoms of SCA/SCD given the fact that all those who participated in the study were taking care of at least 1 child with sickle cell. The signs and symptoms mentioned were as follows; pain (74.19%), swelling of hands or feet (59.95%), jaundice (53.49%), fever (36.56%), headache (26.34%), fatigue (21.52%). These findings are similar to signs and symptoms of sickle cell crisis mentioned as pain, fever, swelling and others including fatigue, jaundice and headache [2]. Children with SCA/SCD have sickle-shaped red blood cells, which block blood flow in tiny blood vessels hence periodic episodes of pain being a more common symptom.

51.88% of the respondents did not know if sickle cell can be cured, 25% disagreed and they were 1.2 times more likely to have knowledge about sickle cell than the remaining 23.12% that agreed (OR = 1.2, p-value = 0.044). Of the 23.12% respondents who agreed, majority believed that God can do it while others said they; had heard from people that it can be cured, believed medicine can work, had seen people get cured. Majority of the participants of the study were believers that is; either Muslims or Christians and may explain why they believed God can cure. However, previous studies show that Hematopoietic stem cell transplantation (HSCT) is the only recognized cure for SCD and has been shown to have an 85–90% success rate in certain pediatric patient groups [13].

A large number of respondents believed that children with sickle cell can cope with life though there were some in disagreement and those that did not know if children with sickle cell can live a normal life. The caregivers who disagreed were 1.6 times more likely to have insufficient knowledge about sickle cell (AOR = 1.6, p-value = 0.001) as compared to the others who agreed. Being caregivers of the children, they are in direct contact monitoring and tracking changes as well as wellness of the child hence they are bound to notice improvements however children with SCD and their families require use of a wide array of coping methods to manage disease-related challenges even though the children can be able to cope with life [14].

36.02% of the respondents in the study were willing to stay with their partners despite the risk of having a child with sickle cell and were 1.8 times more likely to have inadequate information about SCD/SCA (p-value = 0.022, COR = 1.8), 34.68% of the respondents were not sure of the decision to make and were 1.9 times more likely to have less knowledge about SCD/SCA (p-value = 0.012, COR = 1.9) while the remaining 29.30% did not wish to stay in a relationship if there was a risk of having a child with sickle cell. Majority of the participants were married and this maybe a reason to why some might choose to stay.

Majority of the respondents did not know about any social networks for children with sickle cell (p-value = 0.037). Although there is substantial literature documenting the challenges of pediatric SCD for children and their parents, there is limited research identifying how parents prioritize their needs and use their social networks to manage information regarding their child’s SCD in terms of physical and mental health [15]. It is important to ensure that patients are getting accurate information to manage SCD through trusted and supporting social networks. The general attitude of the respondents was poor (72.58%) and only 27.42% had good attitude towards SCD/SCA, this may be influenced by the poor knowledge.

On the basis of our findings, we recommend the following: (1) There is need for the formulation of strategies to encourage male involvement in SCD campaign, (2) It is essential for the inclusion of SCD in existing health education programs both at the community and health center settings/levels to increase knowledge and awareness about SCD and (3) There is need to create awareness about social support networks and engage them more in sickle cell campaigns.

## Conclusion

The findings in this study showed a relatively high level of general awareness about the existence of SCD among caregivers but comprehensive knowledge about the cause and prevention of SCD was low and associated with vast misconceptions though majority of the respondents knew the signs and symptoms of SCD/SCA. Similarly, the general attitude towards SCD/SCA was low and majority did not see a reason why it should influence their marital decisions while others were unsure. There is very low awareness about support services available for sickle cell children among caregivers as majority of them they did not know of any social support group or network that helps children with SCD.

### Electronic supplementary material

Below is the link to the electronic supplementary material.

Supplementary Material 1

## Data Availability

Raw data can be obtained from the corresponding author upon reasonable request.
