# Supplementary material for: Knowledge and attitude towards sickle cell anemia among care givers of paediatric sickle cell patients at a tertiary hospital in Eastern Uganda: a cross sectional study
Source: BMC Res Notes. 2023 Nov 27;16:351. doi: 10.1186/s13104-023-06633-3 (PMC10680200; doi:10.1186/s13104-023-06633-3)
Supplement: Supplementary file 1 — Supplementary Material 1 [file 13104_2023_6633_MOESM1_ESM.pdf]

Supplementary File 1: Study questionnaire

Choose the answer(s) that is appropriate to you in multiple choice questions and fill in blank spaces.

I agree to participate in this study.

A. Yes
B. No

SOCIO-DEMOGGRAPHICS

1. Gender
   A. Female
   B. Male

2. How old are you?
   A. 18 to 30
   B. 31 to 40
   C. 41 to 50
   D. Above 50

3. What is your religion?
   A. Born again
   B. Protestant
   C. Catholic
   D. Muslim
   E. SDA
   F. Others

4. What is your education background?
   A. Primary
   B. Secondary
   C. Tertiary
   D. Uneducated

5. What is your occupation?
   A. Self employed
   B. Formal employed
   C. Unemployed

6. What is your marital status?
   A. Married
   B. Single
   C. Divorced
   D. Others

7. Others, specify:
   ………………………………………..
8. How many children do you have?
   …………………………………….
9. How many have sickle cell disease/ anemia?
   ……………………………………...

KNOWLEDGE ABOUT SCD/SCA

10. Had you ever heard about SCD/SCA before?
    A. Yes
    B. No
11. Do you know how a person can get SCD?
    A. Yes
    B. No
12. How is sickle cell transmitted ?
    A. Parents
    B. Virus
    C. Bacteria
    D. I don’t know others
13. Others, specify;
    ……………………………………

14. Do you have any other person in your family with sickle cell?
    A. Yes
    B. No
    C. I don’t know

15. If yes, who, specify;
    ………………………………..

16. Do you know about sickle cell crisis?
    A. Yes
    B. No

17. Name some of the signs and symptoms of sickle cell crisis.
    A. Pain (joints, chest, abdomen, body)
    B. Fever
    C. Fatigue
    D. Jaundice
    E. Headache
    F. Swelling of feet or arms
    G. Others

    Others, specify;

    ……………………………….

18. To prevent complications, child must take medication and be monitored daily.
    A. Agree
    B. Disagree
    C. I don’t know

19. Sickle cell can only be known by carrying out tests, which of the following tests are done to test sickle cell?
    A. Blood tests
    B. Urine tests
    C. Sugar tests

D. I don’t know
E. Others

ATTITUDE TOWARDS SCD/SCA

20. Sickle cell can be cured.
    A. Agree
    B. Disagree
    C. I don’t know
21. If agree, why?
    ……………………………………

22. Do you think children with sickle cell can cope with life?
    A. Agree
    B. Disagree
    C. I don’t know

23. Would you be willing to stay in a relationship with your partner despite the risk of having a child with sickle cell?
    A. Yes
    B. No
    C. Maybe
24. How do you feel about having a child with sickle cell?
    ……………………………………
25. Are you aware that there are social networks for children with sickle cell and are you in contact with any of these social networks?
    A. Yes I know, and I am in contact with one
    B. Yes I know, and am not in contact with any
    C. No, I don't know of any
    D. others
    Others, specify;………………………….
